# COX-2 gene expression and methylation profile in *Sapajus apella* as an experimental model for gastric adenocarcinoma

**DOI:** 10.1590/1678-4685-GMB-2016-0329

**Published:** 2018-05-14

**Authors:** Danilo do Rosário Pinheiro, Maria Lucia Harada, Rommel Mario Rodriguez Burbano, Barbara do Nascimento Borges

**Affiliations:** 1 Universidade Federal do Pará Universidade Federal do Pará Instituto de Ciências Biológicas Molecular Biology Laboratory BelémPA Brazil Molecular Biology Laboratory, Instituto de Ciências Biológicas. Universidade Federal do Pará, Belém, PA, Brazil; 2 Universidade Federal do Pará Universidade Federal do Pará Instituto de Ciências Biológicas Human Cytogenetics Laboratory BelémPA Brazil Human Cytogenetics Laboratory, Instituto de Ciências Biológicas. Universidade Federal do Pará, Belém, PA, Brazil

**Keywords:** PTGS2, gene regulation, animal model, gastric cancer

## Abstract

Gastric cancer (GC) remains one of the main causes of cancer-related death worldwide. There are two distinct histological types of GC: diffuse and intestinal. The latter is characterized by the presence of pre-neoplastic lesions. One of the most frequently altered enzymes in intestinal GC is COX-2, an important lesion marker. This work aimed to study *COX-2* methylation and expression in N-methyl-N-Nitrosurea (MNU)-induced intestinal GC in six *Sapajus apella* animals. The partial promoter sequence of *S. apella COX-2* gene was obtained and used to identify transcription factors and cis-regulatory element binding sites. The *COX-2* methylation pattern was assessed using Methylation-Specific PCR (MSP), and expression was analyzed by immunohistochemistry (IHQ). A total of 20 samples were obtained. A 675 bp fragment of the *S. apella COX-2* promoter region was obtained, and it was 99.2% and 68.2% similar to *H. sapiens* and *S. boliviensis*, respectively. Similar to humans, several transcription factors and cis-regulatory element binding sites were identified in the *S. apella* sequence. MSP revealed that all samples were methylated. However, IHQ results demonstrated positive COX-2 expression in all pre-neoplastic and tumoral samples. The results suggest that the analyzed fragment is not crucial in *COX-2* regulation of GC in *S. apella*.

Gastric cancer (GC) is the fourth most diagnosed cancer and the second highest mortality rate among all types of cancers worldwide. Its incidence is influenced by several factors, including *Helicobacter pylori* (*H. pylori*) infection, smoking, dietary habits, and host genetic susceptibility (Yan *et al.*, 2013).

Among the many genes involved in gastric carcinogenesis *COX-2* may play an important role. Cyclooxygenase-2 (COX-2) is an inducible enzyme that catalyzes the conversion of arachidonic acid to prostaglandins in response to several inflammatory stimuli. The progression from initial gastric lesions to gastric cancer has been correlated with COX-2 over-expression, suggesting that its activity may be involved in gastric carcinogenesis onset (Li *et al.*, 2012).

It is known that non-human primates are considered a useful model for carcinogenic studies due to their close phylogenic relationship to humans resulting in a great similarity regarding anatomy, physiology, biochemistry, organ systems, and long life span as compared to rodents (Takayama *et al.*, 2008; Costa *et al.*, 2011). Thus, the aim of this study was to evaluate the *COX-2* gene methylation profile and expression in gastric mucosa samples at different pathogenic stages of intestinal gastric cancer in an experimental model developed in primates of the *Sapajus apella* species.

Six adult *Sapajus apella* primates identified with microchips and individually housed in Centro Nacional de Primatas (CENP), Pará State, Brazil, were treated with oral fresh doses of N-Methyl-N-nitrosourea (MNU) (N1517 Sigma-Aldrich, USA) at a dosage of 16 mg/kg body weight, and also received drink water containing MNU in light-shielded bottles daily. As previously described, this concentration was responsible for chemically-induce gastric carcinogenesis (Costa *et al.*, 2011). The animals were fed with fresh fruit, vegetables and commercial food pellets (FOXY Junior Supreme 28% crude protein; PROVIMI, Brazil) and inspected daily and their clinical symptoms were recorded. All the procedures were conducted by veterinarians from CENP. The details of animal welfare and steps taken to ameliorate suffering were in accordance with the recommendations of the Weatherall report, ‘‘The use of non-human primates in research’’. This study was approved by the Ethics Committee of Universidade Federal do Pará (PARECER MED002-13).

All animals were considered healthy at the time of first blood sampling, endoscopy, and ultrasound. This was confirmed by the animals’ behavior as judged by the veterinary check. Periodic endoscopic tests with gastric biopsy and ultrasound (days 0, 90, 120, 300 and 940) were performed throughout the treatment for monitoring. Biopsy samples were subjected to histopathological analysis and sent to the Laboratório de Biologia Molecular at Universidade Federal do Pará (UFPA) for molecular analysis. During the experiment, five animals that developed pre-neoplastic lesions died from intoxication, showing typical symptoms such as confusion, sleepiness, tremor, hyperthermia, diarrhea, vomiting, urinary retention, cutaneous eruptions, and ulcerative oral lesions. They also presented renal, hepatic and respiratory failure and steatosis. Animals suffering and with presumed terminal illness due to adverse side effects were euthanized by intravenous administration of Ketaral (Cetamine chloride, 50 mg/kg), Dormonid (Midazolam, 50 mg/kg) and Methotrimeprazine (Levomepromazine, 50 mg/kg). The surviving animal received Canova treatment and was submitted to a surgical removal of the tumor, being clinically monitored for one year after the end of the experiment. During this period it did not show any complications resulting from the treatments (Costa *et al.*, 2011).

DNA was obtained using the QIAamp DNA Mini Kit (Qiagen) according to the manufacturer’s instructions and quantified using a NanoDrop 1000 Spectrophotometer v3.7 (Thermo Scientific).

The genomic sequence for the *S. apella COX-2* promoter region was obtained from primers designed using conserved regions of the *COX-2* sequence from *Homo sapiens* (GenBank access code: NG_028206) and *Saimiri boliviensis* (GenBank access code: AGCE_01110177). The *COX-2* primer sequences were as follows: sense 5’-GATCACTTCAAAATGAATTCAGGAT-3’ and antisense 5’-GCTACGAAGATAGATTACAGTTATG-3’.

Polymerase chain reaction (PCR) was performed on the normal gastric mucosa samples. The reaction had a final volume of 25 μL containin: 50 ng of template DNA, 10 pM of each primer, 0.20 mM of each dNTP, 2.5 mM MgCl_2_ and 0.5 U *Taq* DNA polymerase (Invitrogen). After PCR, the DNA fragments were sequenced using an ABI 3130 automated sequencer (Life Technologies). The sequencing reaction was performed using the BigDye Terminator v3.1 Cycle Sequencing Kit (Life Technologies).

The obtained sequence was aligned using the BioEdit program (Hall, 2011) with the same sequences used for primer design. Analysis of the promoter region was conducted by comparing the obtained sequence for *S. apella* with the human sequence using the Transcriptional Factor search program (TFsearch) (Heinemeyer *et al.*, 1988) to identify putative transcription factor and *cis*-regulatory element binding sites.

To analyze the methylation pattern of the of *S. apella COX-2* promoter, CpG islands were identified in the promoter fragment using MethylPrimer Express software (Life Technologies). Computational analyses revealed the presence of a 127-bp CpG island containing seven CpG sites located between positions 388 and 514 of the sequenced fragment.

Analysis of the *COX-2* promoter methylation pattern was performed by MSP using selected primers by the MethPrimer software (Li and Dahiya, 2002): COX2MSPMF 5’ AAATAATTAATATAAACTCCG CGAA 3’ and COX2MSPMR 5’ TAGGGAGAGAAATG TTTTAAGGTATAC for the methylated fragment; COX2MSPUF 5’ AAATAATTAATATAAACTCCACA AA 3’ and COX2MSPUR 5’ TAGGGAGAGAAATGTT TTAAGGTATATGT 3’ for the unmethylated fragment. The MSP approach combines the treatment of genomic DNA with sodium bisulfite with PCR amplification using specific primers containing at least one CpG site (Herman *et al.*, 1996). PCR was performed using the Hot Start strategy starting with a cycle of 95 °C for 5 min. After this stage, 40 cycles of 95 °C for 30 s, 51 °C for 30 s and 72 °C for 30 s were repeated, followed by a final extension at 72 °C for 5 min. The PCR product (136 base pairs for both fragments) was loaded onto a 3% agarose gel and visualized using GelRed (Biotium Inc.) staining under UV light.

To detect COX-2 expression in tumor cells the streptavidin-biotin-peroxidase-based immunohistochemical method was performed as described previously (Hsu *et al.*, 1981) with modifications. First, tumor tissue sections (4 mm thickness) were deparaffinized in xylene and rehydrated in a graded series of ethanol. The epitope retrieval was heat-induced followed by incubation with diluted (1:60) COX-2 primary human monoclonal antibody (Zymed/Thermo Fisher, COX2 Monoclonal Antibody COX 229, Catalog Number 35-8200). Histological sections were covered and the slides were incubated at 4-8 ºC for 16 h. A universal peroxidase-conjugated secondary antibody kit was used for the detection system (LSAB + system, DakoCytomation) following the manufacturer’s recommendations. The DAB + System (3,3-diaminobenzidine) (DakoCytomation) was used as the chromogen, following the manufacturer’s recommendations, and haematoxylin was used as the counterstain. Any nuclear stain was considered as a positive result, irrespective of intensity and cytoplasmic staining. Samples were considered positive when 10% or more neoplastic cells were positive for COX-2.

A total of 20 samples was analyzed in this work: six from normal mucosa, six from gastritis, five from atrophic gastritis, two derived from intestinal metaplasia, and one sample of intestinal type of gastric cancer derived from the only *S. apella* that developed the tumor.

When we used the normal mucosa samples, we obtained a 675 bp identical fragment (GenBank accession number KR011346) of the *S. apella COX-2* promoter by PCR. It was 99.2% similar to the *H. sapiens* fragment (4 transitions and 1 transversion) and 68.2% similar to the *S. boliviensis* promoter sequence (134 gaps, 44 transitions and 37 transversions).

We then analyzed the promoter fragment to identify potential transcription factor and *cis*-regulatory element binding sites. We identified several transcription factor binding sites, primarily CdxA, GATA-1 and GATA-2, and *cis*-regulatory elements, including p53 Responsive Elements (P53RE), NF-Y and STAT (Figure 1). Of all the sites examined, only one in the final portion of the fragment was distinct in humans and *S. apella* (CDXA in humans; GATA-1 and GATA-2 in *S. apella*).

**Figure 1:**
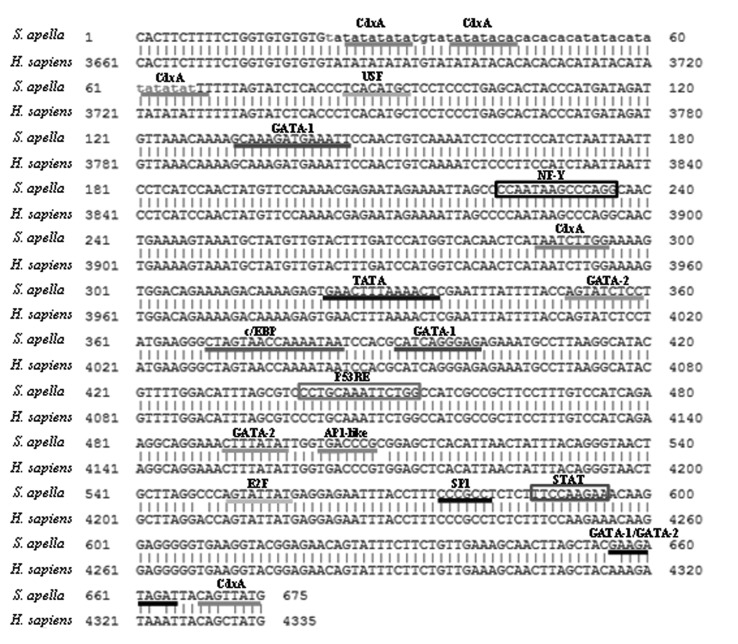
Binding sites in the *S. apella* sequence. Binding sites for regulatory cis-elements (highlighted in boxes) and transcription factors (underlined) in the *S. apella* partial promoter sequence.

When examining the methylation pattern of the *COX-2* promoter, all samples were methylated, regardless of disease state (normal tissue, pre-neoplastic lesions and tumor tissue) **(**Figure 2**).**

**Figure 2:**
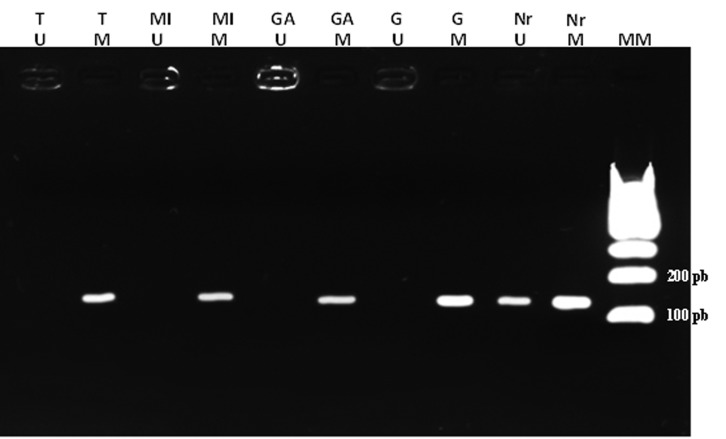
MSP results. Agarose gel (3%) with MSP results. MM: Molecular Marker. M: methylated. U: unmethylated. Nr: Normal tissue. G: Gastritis. GA: Atrophic gastritis (atrophy). MI: Intestinal metaplasia. T: tumor.

Immunohistochemistry was performed to examine COX-2 protein expression in the samples. The results showed that normal samples did not express COX-2, while samples with pre-neoplastic lesions (chronic gastritis, atrophic gastritis and metaplasia) or tumors were positive for COX-2 expression (Figures 3 and 4).

**Figure 3:**
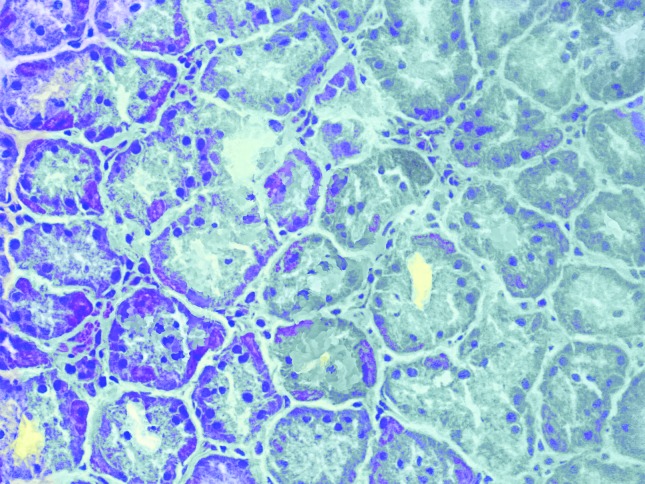
Non-neoplastic gastric mucosa negative for COX-2 immunoreactivity.

**Figure 4:**
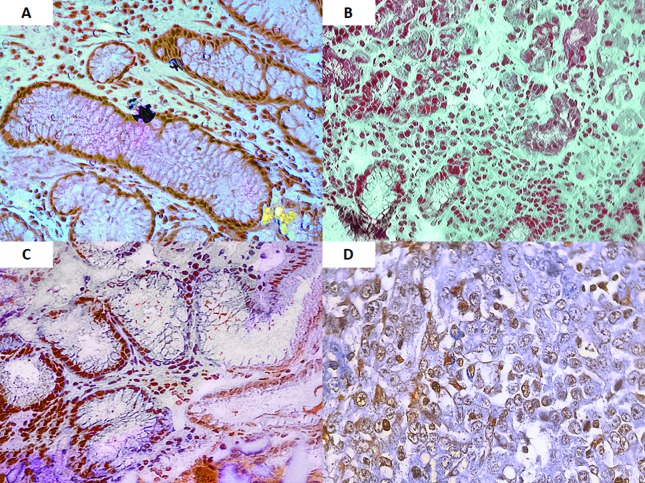
Pre-neoplastic and neoplastic lesions positive for COX-2 immunoreactivity. (A) Chronic gastritis with plasmocytes; (B) Atrophic gastritis (atrophy); (C) Intestinal metaplasia; (D) Tumor

Animal models are a valuable tool to study the origin and molecular mechanisms of cancer and can be used to develop and test new therapeutic strategies, including gastric cancer (Tsukamoto *et al.*, 2007).

Compared to rodents, nonhuman primates are more similar to humans in relation to their genetic evolution, anatomy, physiology, biochemistry and organ system (Puente *et al.*, 2006). In this work, we used the *S. apella* species, an excellent model for biological research purposes, including dental and medical (Gaetti-Jardim Jr *et al.*, 2012), as it can be easily accommodated in Primate Research Centers due to its flexibility, small size, adaptability, opportunism, and because their body size allows for the performance of routine diagnostic tests such as endoscopy, blood sampling and biopsy in the same animal. Besides, our group was the first to establish a gastric carcinogenic model in *S. apella* (Costa *et al.*, 2011).

According to the classification proposed by Lauren (1965), gastric cancer can be divided into two main forms, the intestinal and diffuse types. The MNU-induced lesions in this experiment are consistent with the typical intestinal pre-neoplastic stages. Six animals developed gastritis, and of these, five developed dysplasia, and only two developed metaplasia. Only one monkey survived treatment and developed a tumor. The lesions that appeared during tumor development were similar to those that occur in humans as a multistep process (Lauren, 1965).

Several lines of evidence indicate that inflammatory responses play important roles in cancer development and progression (Kinoshita *et al.*, 2013; Oshima and Oshima, 2013). One of the inflammatory networks involved in gastric carcinogenesis is the cyclooxygenase-2 (COX-2)/prostaglandin E2 (PGE2) pathway. COX-2 is an inducible rate-limiting enzyme for prostaglandin biosynthesis that has an essential role in inflammatory responses (Oshima and Oshima, 2013). Induction of COX-2 expression is found in more than 90% of gastric cancers and is especially triggered by *H. pylori* infection (Echizen *et al.*, 2016). This inflammatory microenvironment promotes the activation of several pathways such as PI3K/Akt/GSK-3β and Notch, which activate the COX-2/PGE2 pathway, leading to gastric tumorigenesis (Thiel *et al.*, 2011; Rivas-Ortiz *et al.*, 2017). In such a way, COX-2 expression is increased in premalignant and malignant lesions, suggesting that this protein plays a role in early gastric carcinogenesis and in tumor progression (Thiel *et al.*, 2011; Cheng and Fan, 2013). Besides being stimulated by an inflammatory process, it is also known that COX-2 expression also plays a role in chemically-induced gastric cancer, using MNU, in rodents (Thiel *et al.*, 2011) and non-human primates (Costa *et al.*, 2011).

COX-2 expression is also regulated by several *cis* elements in its promoter region, such as binding sites for NF-κΒ, and by DNA promoter methylation, as suggested by several studies (Wang *et al.*, 2005; de Maat *et al.*, 2007; Alves *et al.*, 2011), and its expression was associated with several clinicopathological features of gastric carcinogenesis, such as intestinal histological subtype, proximal location, tumor size and advanced clinical stage (Cheng and Fan, 2013).

As the *S. apella COX-2* promoter sequence was not available in the literature, we designed primers from conserved sequences of *H. sapiens* and *S. boliviensis*, a species phylogenetically close to *S. apella.* Upon analysis of the obtained promoter fragment, we observed great similarity between the *S. apella* monkey and the human sequence (99.2%), confirming that it was a conserved portion of the promoter region.

In humans, the *COX-2* promoter region is approximately 1,700 bp long and contains several binding sites for *cis*-regulatory elements and transcription factor binding sites (Wang *et al.*, 2007). In the human promoter, many sites have been identified, including those for C/EBPβ, NFκβ, NF-Y, PEA3, E-box, SP-1, and AP-1, and different combinations of transcription factor binding are responsible for modulating gene expression in different situations (Ratovitski, 2010). Upon further analysis of the amplified *S. apella* region, we identified binding sites for many transcription factors (Figure 1), the most relevant being CDXA, USF, C/EBP and AP-1.

The family of *Cdx* homeobox genes is important for early intestinal epithelial cell differentiation and maintenance and is a major transcription factor that induces the intestinal phenotype, resulting in intestinal metaplasia when expressed in an uncontrolled manner (Barros *et al.*, 2010). In humans, *Cdx* expression is induced in the presence of *H. pylori* and is considered important in gastric carcinogenesis, especially in the intestinal type (Gutiérrez-González and Wright, 2008).

The C/EBP (CCAAT / enhancer binding protein) transcription factor revealed in our analysis has a conserved leucine zipper sequence involved in its homo- and heterodimerization and DNA binding. The presence of the C/EBP binding site in the *COX-2* promoter is well documented, and its binding results in *COX-2* overexpression, including in gastric tumors (Regalo *et al.*, 2006).

We can also highlight AP-1, which plays an important role in carcinogenesis promotion (Santos *et al.*, 2011) and USF (Upstream Stimulating Factor), which belongs to the Helix-Loop-Helix-Leucine Zipper protein family (Corre and Galibert, 2005). Both are known participants in COX-2 transcriptional activation (Santos *et al.*, 2011; Cho *et al.*, 2012).

DNA methylation has also been reported to regulate *COX-2* gene expression and our MSP results revealed a hypermethylated pattern in the *S. apella COX-2* gene promoter. However, a comparison of these results with immunohistochemical analysis did not identify a negative correlation that would confirm the involvement of methylation in *COX-2* gene inactivation. Among the various hypotheses for this lack of correlation between the methylation profile and immunohistochemistry, it is possible that the *S. apella* promoter region analyzed here does not represent a key region for *COX-2* expression (Maat *et al.*, 2007), making further studies on *COX-2* transcriptional regulation necessary.

Our immunohistochemical analysis demonstrated the expression of COX-2 protein in all pre-neoplastic lesions and tumor samples and the lack of expression in normal gastric tissue samples which is confirmed by the results of Lim *et al.* (2000) that COX-2 protein is overexpressed in gastric cancer tissues compared to normal gastric mucosa.

Our results suggest a similarity between the *S. apella* and human *COX-2* promoter sequence, suggesting that *S. apella* is a good animal model for gastric carcinogenesis. Moreover, the lack of correlation between promoter methylation and immunohistochemistry results suggest that this epigenetic mechanism in the analyzed promoter region is not crucial in *S. apella COX-2* regulation.
